# Enhancement of Subjective Quality of Life Following Surgical Intervention in Patients With Drug-Resistant Epilepsy: A Depressive Symptom-Independent Outcome

**DOI:** 10.7759/cureus.57831

**Published:** 2024-04-08

**Authors:** Matheus B Morillos, Daniel T Santos, Debora F Cunha, Ana Paula Gouvêa, Jorge J Bizzi, Carolina M Torres, Marino M Bianchin

**Affiliations:** 1 Neurology, Hospital de Clínicas de Porto Alegre, Porto Alegre, BRA; 2 Nursing, Hospital de Clínicas de Porto Alegre, Porto Alegre, BRA; 3 Hematology, Hospital Nossa Senhora da Conceição, Porto Alegre, BRA; 4 Neurological Surgery, Hospital de Clínicas de Porto Alegre, Porto Alegre, BRA

**Keywords:** beck depression inventory, epilepsy surgery, drug resistant epilepsy, health-related quality of life, quality of life in epilepsy

## Abstract

Purpose: To evaluate the impact of depressive symptoms on the subjective perception of quality of life in patients with drug-resistant epilepsy (DRE) after surgical treatment for seizures.

Methods: A case-control study with DRE patients who received surgical treatment (n=19) and matched non-operated patients (n=23). We assessed the quality of life using the Subjective Handicap of Epilepsy (SHE) scale, alongside measuring depressive symptoms using the Beck Depression Inventory (BDI).

Results: The mean age of the participants was 45 years, with females constituting 52.4% of the patients. A majority (73.8%) had been diagnosed with temporal lobe epilepsy. Those who had undergone surgical intervention showed significantly enhanced performance across all quality-of-life domains on the SHE scale independently of depressive symptoms. The domains of "Work and Activity," "Physical Health," and "Self-Perception" displayed the greatest improvements, with the surgical group's averages outperforming the control group by factors of 1.87, 2.53, and 2.81, respectively. Influential differences impacting the quality-of-life scores included seizure frequency, the quantity of antiepileptic drugs utilized, and the incidence of convulsive seizures.

Conclusion: The findings suggest that surgical control of seizures in drug-resistant focal epilepsy is associated with improvement in quality of life across various domains, independently of the depressive symptoms of the patients.

## Introduction

Epilepsy, a common neurological disorder, is characterized by a persistent predisposition to epileptic seizures and affects over 50 million people globally [1]. It is associated with substantial morbidity and increased functional impairment. The mortality rate among individuals with epilepsy is threefold higher compared to the general population [1,2], with about 80% of epilepsy-related deaths occurring in developing nations [1]. Growing evidence points to a higher incidence of epilepsy in these regions, often attributed, among others, to inadequate perinatal care, increased exposure to infections, and trauma. In Brazil, regional studies indicate a prevalence of epilepsy ranging from 7.8 to 18.6 per 1000 inhabitants [3-6].

Up to 70% of individuals with epilepsy may achieve seizure control with proper diagnosis and antiepileptic drug (AED) treatment [1,2,7]. Nonetheless, about 30% exhibit refractoriness to such pharmacological interventions. This group of patients is diagnosed with drug-resistant epilepsy (DRE) - a condition in which individuals fail to attain seizure control despite trialing at least two adequate therapeutic regimens matched to their specific epileptic syndrome [2]. The prevalence of drug resistance is notably higher in developing countries, exacerbated by insufficient healthcare access, resource allocation discrepancies, and a collective underestimation of epilepsy's impact [1].

Surgical intervention stands as a highly effective option for drug-resistant focal epilepsy patients [2], contingent on meticulous patient selection by specialized multidisciplinary teams within tertiary healthcare settings [8,9]. The principal surgical objective is the excision or disconnection of epileptogenic cerebral tissue, which can lead to seizure freedom in selected cases by up to 80% [2]. The procedure is recognized as cost-effective in developed countries due to the long-term decrease in healthcare expenses [10,11]. Nevertheless, surgical adoption remains limited, especially in resource-constrained regions, due to factors such as insufficient specialist centers, healthcare system barriers, inadequate recognition or knowledge of the procedure's efficacy, and its potential to increase the quality of life in patients with epilepsy [2,12]. Indeed, beyond seizure alleviation, epilepsy surgery outcomes include diminished episode severity, reduced dependence on medication, decreased sudden death risk, and, notably, improved quality of life in these individuals [2,8,13]. In this venue, the evaluation of life quality is pivotal, serving as an essential therapeutic success measure [14]. This aspect is profoundly compromised in patients with drug-resistant focal epilepsy, affecting physical, psychological, and emotional domains, yet the subjective experiences of these patients are often inadequately assessed and depressive symptoms might have a great impact on this matter [15-17].

The objective of our study was to assess the influence of depressive symptoms on the self-perception of quality of life in patients with drug-resistant epilepsy (DRE) who underwent surgical treatment. We also aimed to investigate other factors that may be associated with this impact. To accomplish this, we utilized the Subjective Handicap of Epilepsy (SHE) scale, which is specifically designed for epilepsy and consistently assesses the subjective dimensions of quality of life that are crucial for individuals with epilepsy. Moreover, the SHE scale proves to be a reliable and effective tool for evaluating the quality of life in epilepsy across various settings, including developing regions. Additionally, it is worth mentioning that both the SHE scale and the Beck Depression Inventory (BDI) have been validated for use in Brazilian patients [17,18]. By conducting this research, our goal is to evaluate how epilepsy surgery affects the quality of life in patients with DRE, with a particular focus on understanding how depressive symptoms impact their self-perception of changes in quality of life brought about by surgical interventions for seizure control. 

## Materials and methods

Patients 

This a case-control study that aimed to examine the outcomes of patients over 18 years of age with drug-resistant focal epilepsy regarding quality-of-life changes after surgery and the impact of depressive symptoms on this change. The study included patients who were investigated at the Epilepsy Monitoring Unit at the Hospital de Clínicas de Porto Alegre (HCPA), which is a 786-bed hospital located in Porto Alegre, southern Brazil. The criteria for inclusion were based on the International League Against Epilepsy (ILAE) guidelines for diagnosing drug-resistant focal epilepsy.

The patients were divided into two groups: the operated group and the non-operated group. The operated group consisted of patients who had undergone surgical treatment for their epilepsy, while the non-operated group consisted of patients who were evaluated but had not yet undergone surgery. The reasons for not performing surgery in the non-operated group included patients declining the surgical proposal, risk of damaging eloquent regions near the surgical area, inability to identify the precise epileptogenic focus, and limited resources to cover all surgical candidates in our institution.

All patients underwent a comprehensive evaluation by a multidisciplinary team, including neurologists, neurosurgeons, psychiatrists, and neuropsychologists. They also underwent video-electroencephalography monitoring, 3.0 Tesla brain magnetic resonance imaging, and neuropsychological assessment. Patients with significant intellectual disability, epilepsy of undetermined or generalized origin, psychogenic non-epileptic seizures, or other chronic diseases that could affect their quality of life were excluded from the study. Additionally, patients who did not provide informed consent were not included in the study.

The patients were selected from a database at our Epilepsy Monitoring Unit, which has been collecting data since February 2016. Selected patients were invited to participate in the research and were evaluated during outpatient appointments. The operated group was assessed at an average interval of 4.3 years after surgery, with a range of 0.5 to 7.5 years. The non-operated group was selected to match the clinical and sociodemographic characteristics of the operated group.

This study was approved by the Research Ethics Committee of the Hospital de Clínicas de Porto Alegre and registered in the Brazil Platform under the Certificate of Presentation for Ethical Appreciation (CAAE) number 60750022.4.0000.5327. The study adhered to the ethical principles of the Declaration of Helsinki, and informed consent was obtained from all participating patients.

Quality-of-life assessment

The participants in the study completed the SHE scale to evaluate their quality of life. This instrument is grounded in the World Health Organization's definition of "Handicap" at the time of its development, described as the disadvantage a person encounters while engaging in life roles that diverge from normative societal expectations due to a health condition [18].

The SHE scale, designed by O’Donoghue et al., comprises 32 items across six domains reflecting different aspects of disability: (1) Work and Activity (eight items); (2) Social and Personal Relationships (four items); (3) Physical (four items); (4) Self-Perception (five items); (5) Life Satisfaction (four items); and (6) Change (seven items). Items are rated on a Likert scale from 1 to 5, with the aggregation of subscale scores yielding a total score ranging from 0 to 100 - zero representing the lowest level of satisfaction and 100 representing the highest. Within the "Change" domain, a median score of 50 indicates no perceived change, whereas scores approaching 0 suggest perceived deterioration and those approaching 100 signify perceived improvement [18].

The "Work and Activity" domain evaluates difficulties in employment prospects and retention, including seizure-related job challenges, driving constraints, and impact on leisure activities. The "Social and Personal Relationships" domain assesses the impact of epilepsy on social interactions, familial relationships, and sexual life. The "Self-Perception" domain considers stigma, seizure-related fears, and perceived life control. The "Physical" domain examines the burdens of epileptic symptoms, injuries, and medication side effects. The "Life Satisfaction" domain measures satisfaction within work, leisure, and social interactions. The "Change" domain reflects on personal changes over time concerning epilepsy [18].

The SHE scale has demonstrated reliability, evidenced by a Cronbach's alpha greater than 0.7 for its domains and test-retest reliability with intra-class correlation coefficients between 0.83 and 0.89 (18). Its validity is also established, with satisfactory content validity developed through literature review, expert consultations, and patient input [18]. Construct validity revealed a high discriminative capacity in distinguishing between different levels of disability, and concurrent validity was confirmed through moderate to strong correlations with the Epilepsy Surgery Inventory-55 (ESI-55) instrument [17,18]. The SHE scale has shown sensitivity to detect quality-of-life changes after epilepsy surgery [18].

Moreover, the SHE scale is applicable across cultures, as demonstrated in the Brazilian population, providing consistent, reliable, valid assessments of the quality of life distinct to epilepsy patients [17]. The scale is concise, user-friendly, can be completed in approximately 8 minutes, and is minimally influenced by minor variations in seizure control [18]. It is designed for use in both surgical settings and clinical research and offers predictive value for therapeutic outcomes [17]. This instrument accentuates the subjective dimensions of disability, thus prioritizing the individual's perspective on their quality of life [18]. 

Statistical analysis

Sample size calculations were predicated on existing literature delineating the impact of epilepsy on various quality-of-life domains, as measured by the SHE scale. The domain necessitating the largest sample was "Self-Perception," with a predicted standard deviation of 21.4 in the surgical group, 25.9 in the medically managed group, and a pooled standard deviation of 23.8 [19]. The SHE scale, sensitive to marginal score variances across domains, does not define a threshold for clinically significant change; hence, our research aimed to discern a minimum 20-point distinction among disability domains between groups - a figure posited to represent a clinically meaningful difference. Accordingly, with an alpha level of 0.05 and a power of 80%, a sample size of 36 participants was required (18 per group). Calculations were conducted via PSS Health software, version 1.0.2 [20].

Categorical data were analyzed using the chi-square test, while for non-normally distributed numeric variables, the Mann-Whitney U test was employed. To quantify the relationship intensity between variables, we calculated the effect size of the Mann-Whitney U test via point-biserial correlation, categorizing associations as weak (0.2-0.5), moderate (0.5-0.8), or strong (>0.8). Wilcoxon tests were used for paired samples, with analogous effect size determinations. Statistical significance was acknowledged at p ≤0.05 with a 95% confidence interval. These analyses were executed using Python version 3.6.9, employing the Pandas v. 1.2.5 and SciPy v. 1.7.0 libraries.

Depressive symptoms assessment

The study participants were evaluated for depressive symptomatology employing the Beck Depression Inventory Scale (BDI I), an instrument of international repute for the quantitative assessment of depression severity. This self-report scale is composed of 21 items, each addressing a specific symptom such as affective disturbances, irritability, alterations in sleep patterns, guilt, and thoughts of self-harm. Items are scored on a four-point Likert scale (0-3), reflecting symptom intensity [21]. Gorestein et al. provided a validated Portuguese translation and adaptation of the BDI I for Brazilian studies, proposing an interpretative scoring rubric: scores of 0 to 15 suggest the absence of depression, scores between 15 and 20 indicate mild mood disturbances (dysphoria), and scores exceeding 20 are consistent with depressive pathology [22]. The internal consistency of the instrument evidenced a Cronbach's alpha of 0.81 in non-psychiatric subjects and 0.88 in the psychiatrically diagnosed population [22]. Notably, the BDI I has demonstrated 90% sensitivity and specificity for the identification of depression among epileptic patients [23].

Operated seizure outcome assessment

Seizure outcomes in the operated group were gauged using the Engel Epilepsy Surgery Outcome scale, an established measure of seizure control following surgical intervention for epilepsy [24]. The Engel scale classifies patients into four principal categories, each with specific subcategories reflecting the range of postoperative seizure control. Class I delineates patients who are free of disabling seizures: IA indicates complete seizure remission after surgery; IB signifies the occurrence of non-disabling focal seizures; IC indicates initial disabling seizures with subsequent remission; and ID specifies seizure freedom upon discontinuation of antiepileptic medication. Classes II through IV describe progressively less favorable outcomes, with Class IV denoting no appreciable postoperative seizure control improvement.

Assessment of additional variables

We also evaluated a range of sociodemographic parameters and variables potentially affecting quality of life. These encompassed age, gender, monthly family income, employment, marital status, education level, monthly seizure frequency, number and type of seizures within the last six months, and the count of prescribed AEDs. For participants undergoing surgical treatment, analogous pre-surgical data were retrieved from electronic medical records.

## Results

In the operated group, a total of 23 participants were initially selected. However, four individuals were excluded from the study due to not meeting the eligibility criteria. Specifically, three participants had significant intellectual disabilities, and one participant had a concurrent diagnosis of psychogenic nonepileptic seizures. As a result, the final operated group included 19 participants. In the non-operated group, 23 participants were included, as long as they met the eligibility criteria. These criteria were used to ensure comparability between the two groups. Detailed sociodemographic and clinical data for all participants can be found in Table 1.

**Table 1:** Sociodemographic and clinical data (n = 42) Numerical variables are mean ± SD. Family income in monthly minimum wages.

	Non-operated (n = 23)	Operated (n = 19)	Total (n = 42)	p-Value
Age (mean ± SD)	46.5 ± 11.7	43.2 ± 13.3	45 ± 12.7	0.47
Female gender	13 (56.5%)	09 (47.4%)	22 (52.4%)	0.76
Married or stable union	09 (39.1%)	11 (57.9%)	20 (47.6%)	0.28
Schooling in years (mean ± SD)	7.8 ± 3.3	7.8 ± 3.8	7.8 ± 3.6	0.87
Monthly family income				0.29
<1	04 (17.4%)	01 (05.26%)	5 (11.9%)	-
1 or more and <2	08 (34.8%)	11 (57.89%)	19 (45.2%)	-
2 or more and <3	05 (21.7%)	04 (21.05%)	9 (21.4%)	-
3 or more and <4	05 (21.7%)	02 (10.53%)	7 (16.7%)	-
4 or more	01 (04.3%)	01 (05.26%)	2 (04.8%)	-
Unemployed or retired	16 (69.6%)	12 (63.2%)	28 (66.7%)	0.75
Temporal lobe epilepsy	14 (60.9%)	17 (89.5%)	31 (73.8%)	0.08

The demographic characteristics of the study indicated that the mean age was 45 years, with a slight female predominance (52.4%). Nearly half of the participants (47.6%) were married or in a stable partnership. Educational attainment and family income were generally low within the cohort; a mere 4.7% of participants had attained a bachelor’s degree or an equivalent higher education degree, and 57.1% earned an income below two minimum wages per month. A significant portion (66.7%) of the participants was not engaged in employment at the time of the study. The predominant clinical diagnosis was temporal lobe epilepsy, accounting for 73.8% of the cases, and according to the BDI scale, the majority did not exhibit depressive symptoms. Comparative analysis between two distinct groups yielded no statistically significant differences in the variables considered.

Disability domains in quality of life

**Figure 1:**
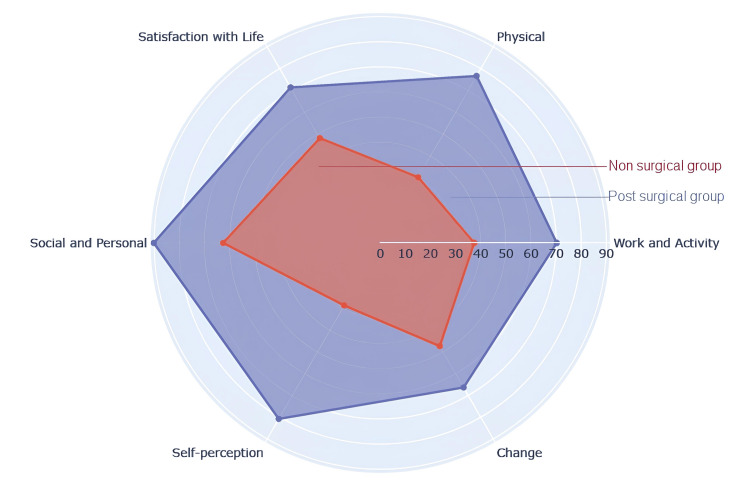
Difference in disability domain scores in quality of life between the operated group (purple) and the non-operated group (orange).

In Table 2, we present the scores acquired for each domain of disability related to quality of life. The non-operated group demonstrated significantly lower scores across all quality-of-life disability domains when assessed with the SHE scale in contrast to the scores from the operated group. Notably, the greatest disparities between non-operated and operated groups were observed in the domains of "Work and Activity," "Physical," and "Self-Perception"; scores from the operated group were 1.87, 2.53, and 2.81 times higher, respectively, indicating a strong effect (r = 0.9) as depicted in Figure 1. Furthermore, the domains labeled "Changes," "Social and Personal," and "Life Satisfaction" showed substantial differences, with the operated group's averages surpassing those of the non-operated group by factors of 1.40, 1.44, and 1.48, respectively, correlating with a moderate effect. 

**Table 2:** Disability domains in SHE scale and depressive symptoms in BDI scale (n = 42) Numerical variables are displayed with mean ± standard deviation. Effect sizes are reported only for significant relations (rank-biserial correlation). SHE = Subjective Handicap of Epilepsy; BDI = Beck Depression Inventory scale.

	Non-operated (n = 23)	Operated (n = 19)	p-Value	Effect size
SHE scale				
Work and Activity (mean ± SD)	37.4 ± 18.7	70.2 ± 11.1	<0.0001	0.9
Physical (mean ± SD)	30.2 ± 20.8	76.6 ± 17.1	<0.0001	0.9
Life-Satisfaction (mean ± SD)	48.1 ± 15.0	71.4 ± 18.7	0.0001	0.6
Social/Personal (mean ± SD)	62.5 ± 21.6	90.1 ± 13.7	<0.0001	0.7
Self-Perception (mean ± SD)	28.7 ± 23.1	80.8 ± 19.5	<0.0001	0.9
Changes (mean ± SD)	47.4 ± 14.8	66.4 ± 19.1	0.001	0.5
BDI				
Score (mean ± SD)	16.1 ± 10.0	10.8 ± 8.3	0.09	

Factors related to quality of life in epilepsy

There was a significant difference between the groups in terms of monthly seizure frequency, the number of AEDs in use, the number of convulsive seizures, and medical consultations in the last six months, as shown in Table 3.

**Table 3:** Comparative data between non-operated group and operated group (n = 42) Numerical variables are displayed with mean ± standard deviation. Effect sizes are reported only for significant relations (rank-biserial correlation).

	Non-operated (n = 23)	Operated (n = 19)	p-Value	Effect size
Monthly frequency of seizures	5.5 ± 5.0	0.0 ± 0.0	<0.0001	0.9
Semiannual convulsive crisis	0.5 ± 1.0	0.0 ± 0.0	0.01	0.3
Anti-seizure number of medications	2.4 ± 0.7	1.2 ± 0.6	<0.0001	0.8
Semiannual outpatient consultation	2.6 ± 2.0	1.2 ± 0.7	0.001	0.6

In the operated group, the preponderance of patients achieved seizure freedom. Within the 30-day period preceding evaluation, no individuals in the operated group experienced focal epileptic seizures. Also, no focal to bilateral tonic-clonic seizures were observed in the preceding six months in this group. Among the 19 individuals in the operated group, 17 attained freedom from incapacitating seizures, classified as Engel Class I. Notably, 14 individuals achieved complete seizure freedom following surgical intervention (Engel Class IA). Two individuals experienced solely non-incapacitating focal seizures after surgery, while one individual endured a single focal to bilateral tonic-clonic seizure upon cessation of AED therapy (Engel Class ID). Additionally, two patients manifested rare, yet debilitating seizures subsequent to the surgical procedure (Engel Class II). One of these patients initially experienced seizure freedom but later exhibited infrequent seizure activity (Engel Class IIA), while the other experienced a reduction in the frequency of debilitating seizures to a rare occurrence over the past two years (Engel Class IIC).

The efficacy of the surgical intervention in terms of seizure management is documented in Table 4. The mean duration between surgical intervention and the subsequent quality-of-life assessment was approximately 4.3 years, with individual spans ranging from 0.5 to 7.5 years. The time intervals corresponding both to the interval from the surgical intervention to the quality-of-life evaluation and to the follow-up are delineated in Table 4. 

**Table 4:** Post-surgical group data and follow-up

Post-surgical group	Epilepsy type	Epilepsy surgery performed	Pathological anatomy analysis	Engel classification	Follow-up (years)
Patient 1	Frontal	Lesionectomy	Inflammation	IA	0.5
Patient 2	Temporal	Standard temporal lobectomy	Hippocampal sclerosis	IA	0.5
Patient 3	Temporal	Standard temporal lobectomy	Pleomorphic xanthoastrocytoma grade II	IA	1.2
Patient 4	Temporal	Lesionectomy	Pleomorphic xanthoastrocytoma grade I	ID	2.0
Patient 5	Frontal	Lesionectomy	Focal cortical dysplasia type II	IA	2.6
Patient 6	Temporal	Standard temporal lobectomy	Hippocampal sclerosis	IIC	3.1
Patient 7	Temporal	Standard temporal lobectomy	Hippocampal sclerosis	IA	3.3
Patient 8	Temporal	Standard temporal lobectomy	Hippocampal sclerosis	IA	4.1
Patient 9	Temporal	Standard temporal lobectomy	Oligodendroglioma grade II	IB	4.1
Patient 10	Temporal	Standard temporal lobectomy	Focal cortical dysplasia type I	IA	4.4
Patient 11	Temporal	Standard temporal lobectomy	Hippocampal sclerosis	IA	4.5
Patient 12	Temporal	Standard temporal lobectomy	Hippocampal sclerosis	IA	4.8
Patient 13	Temporal	Standard temporal lobectomy	Hippocampal sclerosis	IA	6.1
Patient 14	Temporal	Standard temporal lobectomy	Hippocampal sclerosis	IA	6.2
Patient 15	Temporal	Standard temporal lobectomy	Focal cortical dysplasia type II	IA	6.2
Patient 16	Temporal	Standard temporal lobectomy	Hippocampal sclerosis	IB	6.4
Patient 17	Temporal	Standard temporal lobectomy	Grade I ganglioglioma	IA	7.1
Patient 18	Temporal	Standard temporal lobectomy	Neuronal migration disorder	IIA	7.2
Patient 19	Temporal	Standard temporal lobectomy	Inflammation	IA	7.5

In the non-operated group, all individuals experienced a minimum of one focal epileptic seizure within the 30-day timeframe prior to assessment, with a mean occurrence of 5.5 seizures per month. Furthermore, 30.4% of participants within this group sustained at least one seizure with secondary generalization within the preceding six months. Detailed frequency analysis reveals that within this subset of patients, five experienced a mean of one seizure per month, 11 observed two to five seizures per month, four experienced more than five but fewer than 15 seizures per month, and three endured 15 or more seizures per month. Over the course of the last six months, seven individuals manifested at least one focal epileptic seizure with secondary generalization, among whom four had one such event, two experienced two events, and one reported four events.

The integration of depressive symptoms into the model did not yield a significant impact on the correlation between quality-of-life domains and surgical outcomes. Nonetheless, the non-operated cohort exhibited elevated mean scores for depressive symptoms, denoting mild depression (BDI = 16.1), contrasting with the surgically treated cohort, which demonstrated lower mean scores indicative of minimal or no depression (BDI = 10.8). The average number of prescribed anti-seizure medications for the operated group was 1.2 as opposed to 2.4 within the non-operated group. The predominant medications distributed through public healthcare systems included carbamazepine (90.4%), phenobarbital (35.7%), and clobazam (28.6%). Additionally, the non-operated group necessitated an average of 2.6 medical consultations in the past six months, while their surgical counterparts sought consultation approximately 1.2 times during the equivalent period.

Subsequent analyses examining the quality of life after surgery identified notable amelioration in seizure control (both focal and focal to generalized), a decrease in the number of anti-seizure medications utilized, and reduced dependency on outpatient services (as delineated in Table 5). Before surgery, each participant recorded at least one focal seizure monthly, with distribution as follows: four individuals had one seizure, nine had two to five seizures, five experienced more than five but fewer than 15 seizures, and two suffered 15 or more seizures per month. Pertaining to focal seizures with generalization, two patients reported one such event, one had three, and one endured four within the six months preceding surgery. Postoperatively, there were no reported instances of focal epileptic seizures or seizures with generalization in the month leading up to the evaluation. Compared to the period before surgery, the surgical group exhibited an average reduction in semi-annual outpatient visits by 56% and in the use of anti-seizure medications by 45%.

**Table 5:** Comparative data between the period preceding surgical treatment and after the surgery in the operated group (n = 19) Variables are displayed with mean ± standard deviation. Effect sizes are reported only for significant relations (rank-biserial correlation).

	Pre-operating (n = 19)		Operated (n = 19)	p-Value	Effect size
Monthly frequency of seizures	6.3 ± 5.3	0.0 ± 0.0		<0.0001	1.0
Semiannual convulsive crisis	0.5 ± 1.1	0.0 ± 0.0		0.049	1.0
Anti-seizure number medication	2.2 ± 0.7	1.2 ± 0.6		0.002	0.8
Semiannual consultation	2.7 ± 1.2	1.2 ± 0.7		0.001	0.9

A comparative analysis of the non-operated group with baseline pre-surgical data from subjects who later underwent surgery demonstrated no statistically significant differences in seizure control, the number of AEDs prescribed, or the incidence of outpatient visits (Table 6) between these two groups. The preoperative characteristics of the surgical group were comparable to those observed in the non-operated group. This comparison lends support to the hypothesis that surgical intervention had contributed to enhanced quality of life in the operated patients by illustrating that the improvement after surgery is not attributable to baseline differences between the groups.

**Table 6:** Comparative data between the non-operated group and the operated group six months before surgery (n = 42) Numerical variables are displayed with mean ± standard deviation. Effect sizes are reported only for significant relations (rank-biserial correlation).

	Operated group before surgical treatment (n = 19)		Non-operated group (n = 23)	p-Value
Monthly frequency of seizures	6.3 ± 5.3	5.5 ± 5.0		0.51
Semiannual convulsive crisis	0.5 ± 1.1	0.5 ± 1.0		0.57
Anti-seizure medications	2.2 ± 0.7	2.4 ± 0.7		0.20
Semiannual consultation	2.7 ± 1.2	2.6 ± 2.0		0.23

## Discussion

In this study, we have observed a significant improvement in the self-perception of life quality among patients with epilepsy who underwent epilepsy surgery to treat seizures. This improvement was compared with a group of non-operated patients (matched controls) and was evaluated using the SHE scale. The factors associated with this improvement included the frequency of seizures, the frequency of convulsive crises, and the effectiveness of medical treatments. Interestingly, our study found that the improvement in quality of life was not related to depressive symptoms in these patients. This suggests that factors other than depression play a significant role in the increased self-perception of life quality experienced by patients who undergo epilepsy surgery for seizure treatment.

The SHE is a trustworthy and specific tool to evaluate the quality of life in epilepsy in different scenarios. In developed countries, at an epilepsy center in Germany, Buschmann and colleagues evaluated 21 patients with drug-resistant extratemporal epilepsy for quality of life using the SHE scale before and one year after epilepsy surgery [25]. They reported a significant improvement in the "self-perception" and "change" domains of the SHE scale. In Canada, Elliott et al. compared individuals aged 18 to 30 who had undergone epilepsy surgery before the age of 16 with patients with DRE who had not been operated upon [19]. They found significant differences in their scores, noting improvements in the "self-perception" and "physical" domains in the post-surgery group. In a developing country like Brazil, Monteiro et al. performed a transcultural validation study of the SHE scale, observing a significant difference across all disability domains, with lower scores in the pre-surgical group compared to the operated group and the control group [17]. Conversely, in Nigeria, a cross-sectional study assessing the prevalence of epilepsy employed the SHE scale to evaluate the subjective quality of life in 10 epilepsy cases outside the surgical context. Significant differences were found only in the "self-perception" domain in relation to seizure control [26].

In our study, higher quality-of-life values were found in all SHE scale disability domains in the surgical group. The improvement in subjective quality of life may be attributed to better seizure control, as the majority of patients in this group were seizure-free. This improvement was independent of depressive symptoms experienced by the patients. Corroborating our findings, Buschmann et al. also reported that improvements in disability domain scores were positively correlated with seizure control [25]. Even patients who continued to have seizures after surgery exhibited an overall enhancement in quality of life.

Depression is often linked to quality of life in epilepsy, with poorly controlled seizures cited as a major factor associated with depression in this population [27]. Devinsky et al. documented significant improvements in depression symptoms over a two-year follow-up of patients receiving epilepsy surgery [28]. In Brazil, Dias et al. found that patients with drug-resistant temporal lobe epilepsy who had undergone surgical treatment were five times less likely to present with depression postoperatively [29]. However, not all studies align with these findings; Buschmann et al. found no significant changes in depressive symptoms following surgical treatment for epilepsy [25]. Our research did not identify a statistically significant difference in depressive symptoms between the non-operated and surgical groups, suggesting that SHE scale scores may assess broader aspects of well-being that might be in part at least independent of the presence of depression.

The limitations of this study include the small number of patients in each group and the direct analysis of results, which precludes the establishment of causal relationships. This potential bias was mitigated by selecting control groups with homogeneously similar sociodemographic and clinical characteristics. Patient follow-up was influenced by the time period since surgical treatment, as patients operated on in less than one year likely perceive changes related to seizure control more strongly, while those with a longer time since surgery likely present with a lower perception of these effects. Conducted in a public healthcare context within a developing country, the study counters geographical disparities and addresses the therapeutic access challenges faced by the patients. Despite these constraints, the utilization of the SHE scale and BDI, both previously validated for the Brazilian population, provided consistent and reliable insights into the quality-of-life assessments for these patients.

## Conclusions

Patients who underwent surgery for drug-resistant focal epilepsy demonstrated improved scores across all quality-of-life disability domains, as measured by the SHE scale, compared to those with DRE who did not receive surgery. The enhancement in the quality of life observed in the surgical group appeared to be independent of the depressive symptoms reported by patients. Our study does not imply that depressive symptoms or depression are irrelevant to the overall health or quality of life for individuals with epilepsy. Rather, our findings suggest that certain aspects of well-being may be, at least in part, independent of the presence of depression. Additionally, our research highlights the potential value of the SHE scale in developing countries where epilepsy is more prevalent and quality-of-life assessment tools for patients with epilepsy are significantly underused.
